# Differentiating malignant and benign pleural effusion in patients with lung cancer: an ^18^F-FDG PET/CT retrospectively study

**DOI:** 10.3389/fonc.2023.1192870

**Published:** 2023-06-30

**Authors:** Weishan Zhang, Zhe Liu, Xiaoyi Duan, Yan Li, Cong Shen, Youmin Guo, Jian Yang

**Affiliations:** ^1^ PET/CT Department of the First Affiliated Hospital, Xi’an Jiaotong University, Xi’an, Shaanxi, China; ^2^ Radiology Department of the First Affiliated Hospital, Xi’an Jiaotong University, Xi’an, Shaanxi, China; ^3^ Department of Biomedical Engineering, The Key Laboratory of Biomedical Information Engineering of the Ministry of Education, School of Life Science and Technology, Xi’an Jiaotong University, Xi’an, Shaanxi, China

**Keywords:** PET/CT, ^18^F-fluorodeoxyglucose, lung cancer, pleural effusion, pleural metastasis

## Abstract

**Rationale:**

To explore the clinical role of ^18^F-fluorodeoxyglucose positron emission tomography/computed tomography (^18^F-FDG PET/CT) in differentiating malignant pleural effusion (MPE) from benign pleural effusion (BPE) in patients with lung cancer.

**Methods:**

Over a 8-year period, we retrospectively reviewed PET/CT data of lung cancer patients with pleural effusion, with 237 participants enrolled for analysis. The nature of pleural effusion was confirmed using pleural cytology or biopsy. MPE versus BPE comparison and multiple regression analysis were performed. Receiver operating characteristic (ROC) curve analysis was used for evaluating the diagnostic performance.

**Results:**

Of the 237 participants, 170 had MPEs and 67 had BPEs. Compared with BPEs, MPEs had higher pleural SUVmax and thicker pleura and were more common among non-small cell lung cancers, peripheral tumors, and women (p < 0.05). BPEs had larger and higher ^18^F-FDG uptake thoracic lymph nodes and more complications of pneumonia (p < 0.05) than MPEs. Multiple regression analysis was used to identify the pleural SUVmax (odds ratio, OR = 38.8), sex (OR = 0.033), and mediastinal lymphoid node size (OR = 0.86) as independent risk factors for MPEs. The sensitivity, specificity, and area under the ROC curve (AUC) in the combined ROC curve analysis by using the three factors were 95.3%, 95.5%, and 0.989, respectively.

**Conclusion:**

^18^F-FDG PET/CT integrated imaging is an effective non-invasive method for differential diagnosis of MPE in patients with lung cancer. Pleural SUVmax combined with thoracic lymph nodes and sex has high diagnostic value.

## 1 Introduction

Lung cancer is the leading cause of cancer deaths, accounting for 21% of the estimated cancer deaths in the United States in 2022 (1). Pleural effusion (PE) is common in patients with lung cancer. Many of these effusions are malignant, which signify an advanced stage of lung cancer (2); however, some are benign PEs that do not preclude potentially curative surgery. Although pleural invasion adversely affects survival, it is not used extensively during lung cancer staging (3). In a study, including 1279 patients with lung cancer who were undergoing thoracotomy, 4% had an intraoperative pleural effusion of >100 mL that was undiagnosed pre-operatively, and more than 50% of these patients were inoperable (4). It can be difficult to differentiate the malignant and benign effusions because the absence of cancer cells in pleural effusion does not necessarily exclude pleural metastase.

Plain chest radiography and computed tomography (CT) are quick and non-invasive methods for diagnosing malignant PE (MPE). However, they have limited diagnostic sensitivity and specificity (5–7), with CT-based diagnoses missing more than one in three MPEs (5). The performance of magnetic resonance imaging (MRI) is comparable to that of CT; however, MRI lacks a defined role in the initial evaluation of pleural malignancy (8). Ultrasound-guided thoracentesis is the initial procedure of choice for the investigation of suspected MPE, which is confirmed by the presence of malignant cells in the pleural fluid or in the pleural biopsy. However, thoracentesis is an invasive test and can cause complications, such as pneumothorax, hemorrhage, subcutaneous emphysema, traumatic infection, and needle track implantation metastasis. Moreover, its sensitivity is relatively low (approximately 60% (9, 10)), thereby requiring re-examination. Currently, video-assisted thoracoscopic surgery (VATS) is being increasingly performed and has become the gold standard for diagnosing pleural malignancy. VATS provides the best diagnostic performance but is the most invasive diagnostic tool (11).

^18^F-fluorodeoxyglucose positron emission tomography/CT (^18^F-FDG PET/CT) has been widely used for lung cancer characterization and metastasis detection. Maximum standardized uptake value (SUVmax) quantifies ^18^F-FDG uptake by the pleura. Integrated PET/CT imaging enables concurrent functional and anatomical evaluation of the pleura and, therefore, can be used for differential diagnosis of MPE (12–17). To the best of our knowledge, few studies have focused on patients with lung cancer and a pleural effusion. This study explored the clinical role of ^18^F-FDG PET/CT in distinguishing MPE from a benign pleural effusion (BPE) in patients with lung cancer.

## 2 Materials and methods

### 2.1 Study population

**Figure 1:**
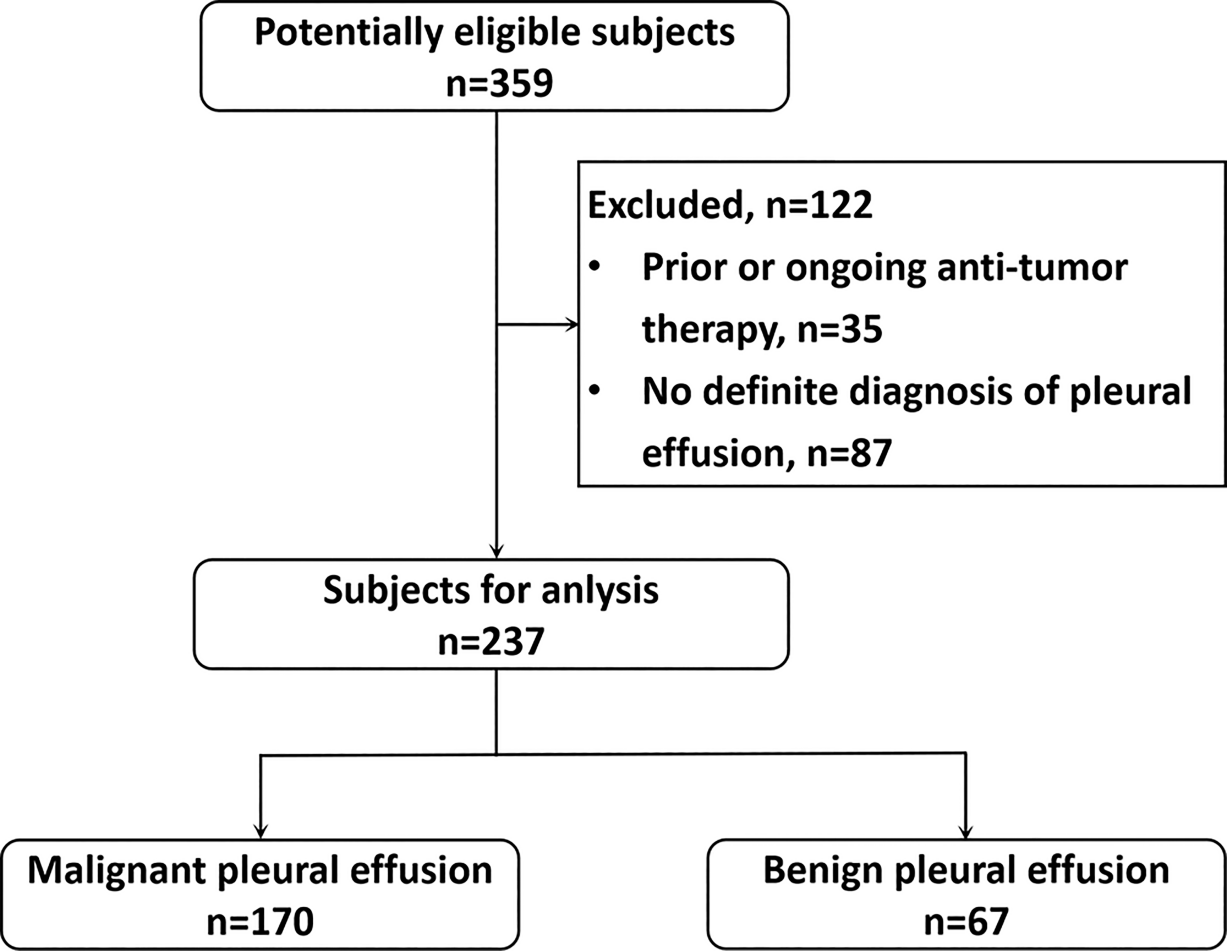
Flow chart of participant selection.

This single-center retrospective study complied with institutional guidelines and regulations and was approved by the ethics committee of the first author’s hospital (No: XJTU1LSK-085). This study was conducted in accordance with the Declaration of Helsinki principles. We retrospectively reviewed the data of patients with lung cancer and pleural effusion who underwent ^18^F-FDG PET/CT from May 2013 to May 2021. This study enrolled 359 participants. Of them, 122 patients with prior or ongoing anti-tumor therapy and indefinite diagnosis of pleural effusion at admission were excluded (**Figure 1**). Finally, 237 patients with primary lung cancer and a pleural effusion were analyzed. Patients without pleural effusion cytology or pleural biopsy and those with prior or ongoing anti-tumor therapy, such as radiotherapy, chemotherapy, surgery, or immunotherapy, were excluded. MPE was diagnosed on the basis of malignant cell confirmation in the pleural fluid or the pleural biopsy. Patients with negative pleural cytology or pleural biopsy results were re-examined. Patients without pleural effusion recurrence 12 months after follow-up (18) were provided a diagnosis of BPE.

### 2.2 ^18^F-FDG PET/CT acquisition

All patients were examined using a PET/CT scanner (Philips Gemini TF 64-PET/CT) consisting of a dedicated germanium oxyorthosilicate full-ring PET scanner and a 64-slice helical CT scanner. After the participants fasted for at least 6 h, their serum glucose levels were measured. The glucose level was less than 200 mg/dL before being administered with 3.7 MBq ^18^F-FDG per kg of body weight (150-300 MBq), followed by resting for 40-60 min in a quiet room. The participants’ plasma glucose levels were measured before FDG administration. The participants were instructed to rest quietly and to refrain from reading and talking during the uptake period. Subsequently, the participants drank 300-600 mL water and emptied the bladder before scanning. Low-dose CT (50 mA, 120 kV), covering the area from the base of the skull to the proximal thighs, was performed for attenuation correction and anatomical localization. Thereafter, a 3-dimensional emission scan was performed, with a scan time per bed position of 1.5 min and 7-10 bed positions, which were acquired according to the body height. PET data were obtained using a high-resolution whole-body scanner with an axial field of view of 18 cm and an overlapping scan of 9 cm. The average axial resolution was 4.2 mm full width at half maximum (FWHM) in the center. The average total PET/CT examination time was approximately 15 min. The acquired PET data were reconstructed iteratively with attenuation correction, and the reconstructed PET images (CT attenuation-corrected, CTAC) were obtained and reoriented into axial, sagittal, and coronal views. The data were transferred to the Philips Extended Brilliance Workstation (EBW) for image analysis and interpretation.

### 2.3 PET/CT interpretation

The PET/CT images were reviewed by two experienced physicians working in the PET/CT center for more than 10 years. Both physicians were blinded to the final diagnosis of pleural effusion. The PET and CT integrated images were automatically obtained using Philips EBW. ^18^F-FDG PET/CT imaging was interpreted by visual interpretation, and by combining the degree and form of the pleura and lymph nodes ^18^F-FDG uptake on PET imaging with the morphologic feature on CT imaging. In cases of discrepancy regarding PET/CT findings, a final consensus was obtained on all imaging findings after mutual discussion between them.

The degree of ^18^F-FDG uptake in pleural region and lymph nodes were compared with background activity, and higher than that in the surrounding soft tissues were regarded as positive. The semiquantitative parameter SUVmax was obtained by a circular region of interest (ROI) with proper diameter, placed manually over the corresponding area in the cross-sectional slice of attenuation-corrected emission images. ^18^F-FDG uptake of the pleura was measured by manually drawing ROI on PET and CT registered images slice by slice, and the maximal SUVmax was selected to represent the pleural ^18^F-FDG uptake.

The pleura thickness was measured using axial CT images. In patients with no obvious pleural thickening, nodules, or masses, the site of the highest ^18^F-FDG uptake on PET images was used for CT image measurement. The highest ^18^F-FDG uptake lesion was selected to measure the pleural thickness and SUV if multiple pleural lesions were present. SUVmax of the primary tumor was measured. For lymph nodes, the nodes with the highest SUVmax in the hilar and mediastinal regions were delineated for measurement, respectively. The tumor-pleura distance was defined as the shortest distance between the tumor and pleura, and the participants were divided into three groups on the basis of the tumor-pleura distance (**Table 1**). CT sign identification was based on the consensus between the two physicians. Lung-cancer-related pulmonary complications in the participants included obstructive pneumonia, atelectasis, and consolidation.

**Table 1:** Comparison between benign and malignant pleural effusions in patients with lung cancer.

Variables	Comparison between groups of pleural effusion		
	Benign (n = 67)	Malignant (n = 170)	*p*
**Age**	61.5 ± 12.1	60.9 ± 10.1	0.510
**Gender**			<0.001
**Male(n = 157)**	59 (86.8%)	98 (57.1%)	
**Female(n = 80)**	8 (13.2%)	72 (42.9%)	
**Pathology**			<0.001
**NSCLC (n = 215)**	49 (73.7%)	166 (97.8%)	
**SCLC (n = 22)**	18 (26.3%)	4 (2.2%)	
**Lung tumor location**			0.003
**Peripheral (n = 111)**	21 (31.4%)	90 (52.9%)	
**Central(n = 126)**	46 (68.6%)	80 (47.1%)	
**Complication of pneumonia**			0.008
**Yes (n = 134)**	47 (71.1%)	87(51.1%)	
**No (n = 103)**	20 (29.8%)	83 (48.9.3%)	
**Pleural effusion location**			0.590
**Ipsilateral (n = 155)**	50 (74.6%)	135 (79.4%)	
**Bilateral (n = 45)**	14 (20.8%)	31 (18.2%)	
**Contralateral (n = 7)**	3 (4.4%)	4 (2.4%)	
**Tumor-pleura distance(mm)**	2.6 ± 6.0	3.7 ± 7.1	0.086
**Lung tumor size (mm)**	50.3 ± 18.6	38.3 ± 24.0	<0.001
**Lung tumor SUVmax**	9.0 ± 3.3	9.5 ± 3.5	0.383
**Pleural thickness (mm)**	2.1 ± 0.9	8.0 ± 5.6	<0.001
**SUVmax of pleura**	1.7 ± 0.5	5.6 ± 3.6	<0.001
**Mediastinal LN size (mm)**	21.8 ± 13.1	10.9 ± 5.6	<0.001
**Mediastinal LN SUVmax**	6.8 ± 2.8	5.7 ± 3.7	0.002
**Hilar LN size (mm)**	20.2 ± 12.1	10.6 ± 7.4	<0.001
**Hilar LN SUVmax**	6.8 ± 3.0	5.8 ± 3.5	0.005

NSCLC, Non-small cell lung cancer; SCLC, small cell lung cancer; SUVmax, maximal standardized uptake values; LN, lymph node.

### 2.4 Statistical analysis

Continuous variables are expressed as mean ± standard deviation, and categorical variables are presented as frequencies and percentages. The independent *t*-test was used for normal data, and a nonparametric test was used for non-normal data. A chi-square test was used for analyzing classified data. Independent risk factors were determined, and odds ratios (ORs) were calculated using the multiple logistic regression analysis. Receiver operating characteristic (ROC) curve analysis was used for evaluating the diagnostic efficiency of PET/CT in distinguishing MPE from BPE by using the independent risk factors. Sensitivity and specificity were calculated, and the area under the curve (AUC) values were determined. SPSS Statistics for windows (24.0, USA) and Medcalc (13.0, MedCalc Software Bvba, Ostend, Belgium) were used for data analysis. Statistical significance was defined as two-tailed p < 0.05.

## 3 Results

### 3.1 Patient characteristics

All patients (age: 28-88 years, male/female: 157/80) had biopsy-based primary lung cancer diagnosis, with the following pathology types: 22 small cell lung cancers and 215 NSCLCs (adenocarcinoma 152, squamous 32, and unspecified NSCLC 31). Of the 237 patients, 170 had a diagnosis of pleural metastasis made on the basis of pleural effusion cytology by using thoracocentesis (n = 92), pleural biopsy (n = 25), thoracoscopic surgery (n = 21), and with two or more tests (n = 32). Negative histology was confirmed in 67 patients by using thoracoscopic surgery (n = 21), pleural biopsy (n = 12), and pleural effusion cytology (n = 34), with negative findings on chest CT after > 1-year follow-up.

### 3.2 Comparison between MPE and BPE

**Figure 2:**
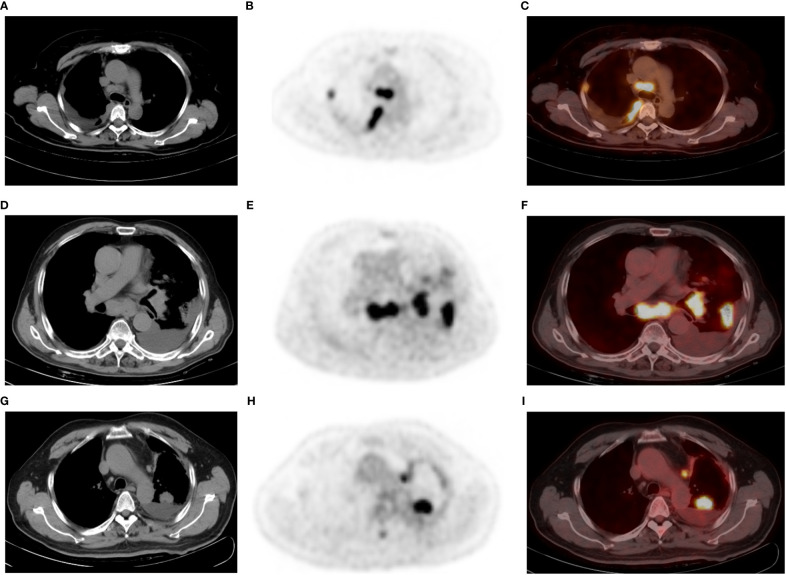
A 52-year-old man with poorly differentiated lung adenocarcinoma and right-sided malignant pleural effusion **(A–C)**. Axial CT **(A)** revealing effusion in the right pleural cavity, and axial ^18^F-FDG PET **(B)** and fused PET/CT **(C)** revealing nodular ^18^F-FDG uptake (SUVmax 4.4) in the same pleural region. Thoracentesis-based pathology confirmed MPE caused by metastatic adenocarcinoma. A 77-year-old man with lung adenocarcinoma and left-sided BPE **(D–F)**. Pleural fluid cytologic findings were negative, without recurrence of pleural abnormalities on follow-up. Axial CT **(D)** revealing a left peripheral lung cancer with left pleural effusion, as well as pneumonia, mediastinal, and left hilar lymphadenopathy. Axial PET **(E)** and fused PET/CT **(F)** revealing hypermetabolic areas in the tumors without abnormal FDG uptake in the pleura. A 65-year-old man with left lung adenocarcinoma and left-sided MPE **(G–I)**, who did not have an enlarged lymph node in the mediastinum or hilar region. Axial CT **(G)**, PET **(H)** and fused PET/CT **(I)** revealing a left PE, lung mass (SUVmax: 13.9), and mediastinal pleural thickening (thickness: 5.6 mm and SUVmax: 4.1). PE cytology confirmed MPE. Note that the largest thoracic lymph node of this patient is located next to the aortic arch with a short diameter of 7 mm and SUVmax 6.5.

Significant differences were observed between MPEs and BPEs in the following characteristics: sex, primary lung tumor (pathology, location, and size), complications of pneumonia and mediastinal and hilar lymph nodes (size and SUVmax), and thickness and SUVmax of pleura. No significant differences in age, pleural effusion location, tumor-pleura distance, and ^18^F-FDG uptake of lung tumor were observed between MPEs and BPEs (**Table 1**). Of the 215 NSCLCs, 166 had MPE. 72 women patients were MPEs (68 adenocarcinomas and only 4 SCLCs). MPEs were more common among NSCLCs and women (p < 0.01), and exhibited higher pleural ^18^F-FDG uptake values (SUVmax: 5.6 vs 1.7) and thicker pleura (8.0 mm vs 2.1 mm) than BPEs. BPEs had larger mediastinal and hilar lymph nodes and had higher ^18^F-FDG uptake than MPEs (p < 0.001). Of all the 237 subjects, 124 had the lymph nodes < 10mm, which were all NSCLC, and 86.3% (107/124) of them were the MPE. The typical PET/CT imaging features of MPE and BPE are displayed in **Figure 2**. Receiver operating characteristic (ROC) curve analysis in our study determined pleural SUVmax 2.5 as the cutoff value, and eighteen MPEs were false negative on PET based on the cut-off value, 12 of them did not present pneumonia or enlarged lymph nodes in mediastinal or hilar regions. Only four false positive results were obtained in patients with a diagnosis of tuberculous pleurisy. The multiple regression analysis identified pleural SUVmax (OR = 38.8), sex (OR = 30.3), and mediastinal lymphoid node size (OR = 0.86) as independent risk factors of MPE.

### 3.3 ROC curve analyses

ROC curve analyses were performed to determine the diagnostic value of the independent risk factors of MPE. The AUCs of pleural SUVmax, mediastinal lymphoid node size and sex were 0.968 (95% CI: 0.937-0.986), 0.765 (95% CI: 0.706-0.817), and 0.652 (95% CI: 0.588-0.713), respectively. The comprehensive AUC of the three independent risk factors was 0.989 (95% CI: 0.966-0.998), and the sensitivity and specificity were 95.3% and 95.5%, respectively.

## 4 Discussion

This study retrospectively assessed the PET/CT data of patients with lung cancer and a pleural effusion. Compared with BPEs, MPEs exhibited a higher pleural SUVmax and thicker pleura and nodules. Women and NSCLCs exhibited a higher prevalence of MPE than men and SCLCs. BPE was often detected in patients with central lung cancer and the complications of obstructive pneumonia and larger thoracic lymph nodes. The pleural SUVmax, sex, and mediastinal lymphoid node size were the independent risk factors of MPE. Based on a relatively large sample size, the study validates the diagnostic value of PET/CT in differentiating malignant and benign pleural effusion (PE) in patients with lung cancer. The increased clinical use of ^18^F-FDG PET/CT and the frequent development of pleural effusions in lung cancer patients make it important to determine the significance of PET/CT imaging characteristics in the assessment of PE. For the first time, female was reported as a risk factor for MPE; and the study found that if there are no enlarged lymph nodes in the hilar or mediastinal, the cause of pleural effusion is more likely to be pleural metastases. We believe that our study makes a significant contribution to the literature and these results may serve as a reliable method to diagnose and classify pleural effusion in lung cancer patients.

Pleural metastasis is a common cause of pleural effusion in patients with lung cancer. Morphological imaging is of limited use in the assessment of pleural effusion conditions. On CT imaging, pleural effusion is characterized by pleural thickening ≥1 cm that is nodular, circumferential, or extending onto the mediastinal surface (19). The micro pleural lesions detected during surgery or thoracoscopy are mostly neglected during CT examination (20). The ^18^F-FDG PET/CT is promising in the detection of malignant lesions with abnormal glucose uptake. MPEs often exhibit focal ^18^F-FDG uptake in the parietal pleura, and the diagnostic accuracy can be improved using the semi-quantitative analysis of SUV measurement. The sensitivity and specificity of MPE detection by using PET/CT are approximately 89%-100% and 67-94%, respectively (14, 21–23). The results of this study are consistent with those of previous studies.

In this study, MPE diagnosis was satisfactory, with an AUC of 0.989. Only four false positive results were detected using the cut-off value of pleural SUVmax 2.5 in patients with a diagnosis of tuberculous pleurisy, which is characterized by pleural thickening and increased uptake of radionuclides on PET/CT imaging (16). Tuberculous pleurisy is difficult to differentiate from pleural metastases in patients with lung cancer. Liao et al. (24) reported 33 lung cancer cases with pleural effusion (27 MPEs and 6 BPEs), with only one case with tuberculous pleurisy being false positive on PET/CT. Histocytological confirmation should be performed to distinguish tuberculous pleurisy from malignant effusions.

In this study, 18 MPEs were false negative on PET. Of these, 12 MPEs had no obstructive inflammation, consolidation, atelectasis, or enlarged lymph nodes in the mediastinal or hilar region which are the common indirect causes of pleural effusion in patients with lung cancer. A study indicated that pleural effusion is caused by direct and indirect mechanisms (25). The direct mechanism mainly refers to pleural invasion, whereas the indirect mechanism includes tumor involvement of mediastinal lymph nodes, lymphangitic metastasis, bronchial obstruction, pneumonia, and uncontrolled heart, liver, or kidney disease. We suggest that if no indirect causes of pleural effusion are identified in patients with lung cancer, pleural metastasis should be considered even if the ^18^F-FDG uptake of pleura does not increase significantly. Moreover, further examination is recommended for the accurate assessment of pleural effusion.

The causes of pleural effusion in SCLCs and NSCLCs differ. Pleural effusion in SCLCs is caused by indirect mechanisms, whereas that in NSCLCs is caused by direct mechanisms. Ryu et al. (25) reported that 12.3% of patients with SCLCs and pleural effusions had pleural metastases, which was much lower than that in patients with NSCLCs (87.8%) (3). Our results demonstrated that 166 of 215 NSCLCs (77.2%) were MPEs, and 68 of 72 female MPEs (94.4%) were adenocarcinomas. In this study, the incidence of MPE was higher in women than in men, and sex was an independent risk factor for MPE. This may be attributed to the higher proportion of adenocarcinoma in women than in men. Another contributing factor to the higher rate of female MPE may be the estrogen, which can increase the proliferation, migration, and invasion of lung cancer (26, 27).

In our study, BPE had a larger mediastinal lymph node than MPE. This maybe because the lymph node involvement causes obstruction of lymphatic circulation, resulting in BPE. Ryu et al. (25) reported that 69 of 74 patients with SCLCs and pleural effusions presented with bulky lymphadenopathy in the mediastinum or hilum, with only one of them being MPE. For NSCLC, the results are not entirely consistent. Li Y (28) reported that there was no significant difference in the incidence of lymph node enlargement between BPE and MPE, and the SUVmax of node was higher in the malignant group (MPE vs BPE= 6.0 vs 4.9, p=0.032). The reason for the inconsistency between these results and ours may be due to the different methods used to delineate the lymph nodes. As they only selected the short-axis diameter of lymph node larger than 10 mm, resulting in the small lymph node not being included as part of the analysis. Most of the pleural effusion patients with the small lymph nodes are malignant. In our results, of all the 237 included subjects, 124 had the lymph nodes < 10mm, which were all NSCLC, and 86.3% (107/124) of them were the MPE. Moreover, the small lymph nodes often have lower SUVmax. To some extent, this difference reflects the importance of our findings that if lung cancer patient does not have enlarged lymph nodes, the cause of pleural effusion is more likely to be pleural metastases.

Our study has some limitations. We enrolled only patients with pathological results. In patients with minimal pleural effusion (effusion thickness of< 10 mm on CT images (29)), cytological diagnosis by using thoracentesis was difficult. Most of the minimal pleural effusion patients were excluded because of unavailable pathological results, and there were only seven patients were enrolled as diagnosed using thoracoscopic surgery. Second, the number of patients with SCLC was relatively less than NSCLC; moreover, we did not have data on estrogen levels in this cohort of patients as it was a retrospective study. More relevant research data need to be augmented in future studies to further confirm the findings.

In conclusion, ^18^F-FDG PET/CT integrated imaging is an effective non-invasive method for the differential diagnosis of MPE in patients with lung cancer. Increased pleural ^18^F-FDG uptake and pleura thickening suggest pleural metastases. Pleural glycolysis combined with thoracic lymph nodes and sex has high diagnostic value. The increased clinical use of ^18^F-FDG PET/CT and frequent pleural effusions in patients with lung cancer increase the importance of PET/CT characteristic usage in pleural effusion assessment.

## Data availability statement

The original contributions presented in the study are included in the article/supplementary material. Further inquiries can be directed to the corresponding authors.

## Ethics statement

The studies involving human participants were reviewed and approved by the ethics committee of the First Affiliated Hospital of Xi’an Jiaotong University (No: XJTU1LSK-085). Written informed consent for participation was not required for this study in accordance with the national legislation and the institutional requirements.

## Author contributions

(I) Conception and design: WZ, ZL and JY. (II) Administrative support: ZL. (III) Provision of study materials or patients: WZ, XD, YL and JY. (IV) Collection and assembly of data: WZ and ZL. (V) Data analysis and interpretation: WZ and ZL. (VI) Manuscript writing: WZ, ZL and JY. (VII) Final approval of manuscript: All authors.
